# KB-R7943 reduces 4-aminopyridine-induced epileptiform activity in adult rats after neuronal damage induced by neonatal monosodium glutamate treatment

**DOI:** 10.1186/s12929-017-0335-y

**Published:** 2017-05-09

**Authors:** Mariana Hernandez-Ojeda, Monica E. Ureña-Guerrero, Paola E. Gutierrez-Barajas, Jazmin A. Cardenas-Castillo, Antoni Camins, Carlos Beas-Zarate

**Affiliations:** 1 Laboratorio de Biología de la Neurotransmisión, Edificio de Posgrado, Departamento de Biología Celular y Molecular, CUCBA, Universidad de Guadalajara, Km 15.5 Carretera a Nogales, Camino Ing. Ramón Padilla Sánchez Km 2, Zapopan, Jalisco Mexico 45221; 2 Unitat de Farmacologia i Farmacognòsia, Institut de Neurociencias, Facultat de Farmàcia, Universitat de Barcelona, Barcelona, Spain; 3 Biomedical Research Networking Center in Neurodegenerative Diseases (CIBERNED), Madrid, Spain

**Keywords:** Monosodium glutamate, NCX1-3, KB-R7943, Seizures, Hippocampus, Entorhinal cortex

## Abstract

**Background:**

Neonatal monosodium glutamate (MSG) treatment triggers excitotoxicity and induces a degenerative process that affects several brain regions in a way that could lead to epileptogenesis. Na^+^/Ca^2+^ exchangers (NCX1-3) are implicated in Ca^2+^ brain homeostasis; normally, they extrude Ca^2+^ to control cell inflammation, but after damage and in epilepsy, they introduce Ca^2+^ by acting in the reverse mode, amplifying the damage. Changes in NCX3 expression in the hippocampus have been reported immediately after neonatal MSG treatment. In this study, the expression level of NCX1-3 in the entorhinal cortex (EC) and hippocampus (Hp); and the effects of blockade of NCXs on the seizures induced by 4-Aminopyridine (4-AP) were analysed in adult rats after neonatal MSG treatment. KB-R7943 was applied as NCXs blocker, but is more selective to NCX3 in reverse mode.

**Methods:**

Neonatal MSG treatment was applied to newborn male rats at postnatal days (PD) 1, 3, 5, and 7 (4 g/kg of body weight, s.c.). Western blot analysis was performed on total protein extracts from the EC and Hp to estimate the expression level of NCX1-3 proteins in relative way to the expression of β-actin, as constitutive protein. Electrographic activity of the EC and Hp were acquired before and after intracerebroventricular (i.c.v.) infusion of 4-AP (3 nmol) and KB-R7943 (62.5 pmol), alone or in combination. All experiments were performed at PD60. Behavioural alterations were also recorder.

**Results:**

Neonatal MSG treatment significantly increased the expression of NCX3 protein in both studied regions, and NCX1 protein only in the EC. The 4-AP-induced epileptiform activity was significantly higher in MSG-treated rats than in controls, and KB-R7943 co-administered with 4-AP reduced the epileptiform activity in more prominent way in MSG-treated rats than in controls.

**Conclusions:**

The long-term effects of neonatal MSG treatment include increases on functional expression of NCXs (mainly of NCX3) in the EC and Hp, which seems to contribute to improve the control that KB-R7943 exerted on the seizures induced by 4-AP in adulthood. The results obtained here suggest that the blockade of NCXs could improve seizure control after an excitotoxic process; however, this must be better studied.

**Electronic supplementary material:**

The online version of this article (doi:10.1186/s12929-017-0335-y) contains supplementary material, which is available to authorized users.

## Background

Excitotoxicity is a complex process in which several mechanisms lead to intracellular Ca^2+^ overload and cell death observed in various neuropathological conditions [1–3]. The most common trigger mechanism of the excitotoxicity in neurons is the overactivation of receptors for the excitatory neurotransmitter glutamate (Glu) [1, 4], mainly the ionotropic receptor selectively activated by N-Methyl-D-Aspartate (NMDA), whose overactivation causes a prolonged increase in the intracellular Ca^2+^ concentration ([Ca^2+^]_i_) and the subsequent proteolysis of diverse proteins, including receptors and exchangers among others [5, 6]. Monosodium glutamate (MSG) is probably the broad-spectrum glutamate analogue most used to study excitotoxic neuronal damage [7, 8]. Neonate animals are more susceptible to MSG toxic effects, and repeated systemic administration of MSG at neonatal age produces neuronal death in the short term [9, 10] and several degenerative changes in the long term [11–14]. The expression levels and functionality of numerous enzymes [15, 16], receptors [9, 10, 17, 18] and transporters [14, 19] are modified by neonatal MSG treatment. Recently, a significant increase in the expression level of mRNA encoding the sodium/calcium (Na^+^/Ca^2+^) exchanger type 3 (NCX3) was reported in the hippocampus (Hp) twenty hours after cessation of treatment [19], but it is unknown if neonatal MSG treatment has any long-term effects on this exchanger.

On the other hand, sodium/calcium (Na^+^/Ca^2+^) exchangers (NCXs) show a better capacity to transport Ca^2+^ than the plasma membrane Ca^2+^-ATPase (PMCA) [20], and they are essential for maintaining [Ca^2+^]_i_ homeostasis [21]. In mammals, these exchangers belong to the solute carrier family 8 (Slc8a) and are represented by three independent genes that encode for NCX1 to 3 proteins, which share close to 70% of their amino acid sequence [22–24]. NCX expression is differentially regulated at the transcriptional level [25]. Increases in [Ca^2+^]_i_ upregulate NCX1 and NCX3 expression, while NCX2 expression is downregulated [26]. Recently, it has been reported that early effects of nerve growth factor (NGF) include upregulation of NCX1 and NCX3 expression and downregulation of NCX2 expression; all these effects depend on the p38 signalling pathway activation and are related to increases in the CREB1 transcription factor binding to the promoter of NCXs [27]. NCXs are expressed in a tissue-specific manner, where NCX1 is found in several tissues including the brain, and NCX2 and NCX3 are mainly expressed in the brain and skeletal muscle [28, 29]. In the brain, the expression level of NCX1 and NCX2 is higher than the expression level of NCX3 during development [30, 31], where NCX1 and NCX3 are predominantly located in neurons, while glial and endothelial cells express all three exchangers [32, 33]. NCXs exchange 3 Na^+^ for 1 Ca^2+^ and operate bidirectionally, in a forward mode expelling Ca^2+^ and in a reverse mode introducing it [21, 34]. Rises in [Ca^2+^]_i_ activate the forward mode of the exchangers, and rises in [Na^+^]_i_ activate the reverse mode [34, 35]. Interestingly, during the excitotoxic process, the Na^+^ influx promoted by Glu receptors could activate the reverse mode of NCXs and contribute to Ca^2+^ overload, potentiating the damage [36, 37]. Moreover, NCX1 and NCX3 in reverse mode could contribute to epileptogenesis [38, 39], and their blocking improves the control of seizures in different animal models of epilepsy [38, 39]. KB-R7943 (2-[2-[4-(4-nitrobenzyloxy)phenyl]ethyl]isothiourea methanesulfonate) preferentially blocks NCXs in reverse mode, is 3-fold more effective on NCX3 [40], and exerts a neuroprotective effect in the excitotoxic process [41, 42]. Furthermore, KB-R7943 orally administered prevents seizures induced by pilocarpine and pentylenetetrazole (PTZ) in adult animals [38, 39]. However, high doses of KB-R7943 may interact with other transporters and reduce the beneficial effects describe above [43].

Because neonatal MSG treatment modifies several GABAergic markers and reduces GABA-positive cell density in the Hp of adult male rats [14, 15], seizure susceptibility was thought to be altered by MSG treatment. Therefore, preliminary results published by our group showed that neonatal MSG treatment increases the susceptibility to seizures induced by 4-aminopyridine (4-AP), reaching more severe convulsive signs with lower doses than in intact animals [44]. Historically, 4-AP has been known as an organic compound that blocks A- and D-voltage-gated K^+^ channels [45, 46], avoiding neuronal repolarization, prolonging the depolarizing phase of the action potential, and promoting vesiculated release of neurotransmitters [47]. Over the years, both *in vitro* and *in vivo* studies have demonstrated that 4-AP could induce electrographic [48–50] and convulsive seizures [51, 52], respectively. Seizures induced by 4-AP correspond to limbic seizures and have been mainly related to strong increases in Glu release [53, 54]. The ionic imbalance produced by 4-AP also may affect the functionality of NCXs; however, this hypothesis has been poorly studied after an excitotoxic process and during epileptogenesis, but we addressed the issue here.

Epilepsy is a neurological disorder that ails 65 million people worldwide^,^ who suffer from recurring and spontaneous seizures [55]. Although almost 20 antiepileptic drugs are available on the market and several more drugs are in preclinical phases [56], 30 to 40% of epileptic patients present pharmacoresistant seizures [57, 58]. Therefore, it is important to continue the characterization of drugs, such as KB-R7943, with possible antiepileptic effects. In the present study, we analysed the expression level of NCX proteins in the entorhinal cortex (EC) and Hp of male adult rats after neonatal MSG treatment. In addition, behavioural and electroencephalographic activities were recorded after intracerebroventricular (i.c.v.) infusion of KB-R7943 and 4-AP, both alone and in combination, in male adult rats neonatally treated with MSG.

## Methods

### Animals and neonatal treatment

Pregnant Wistar rats were used and kept under optimal environmental conditions in separate cages with water and food provided *ad libitum*, 12/12 h light/dark cycles and the room temperature at 23–25 °C. At dawn, all litters were adjusted to eight offspring per female. Only males were used for this study. Monosodium glutamate (MSG: 4 g/kg body weight; Cat. G1626, Sigma-Aldrich, MO, USA) was subcutaneously (s.c.) administered on postnatal days (PD) 1, 3, 5, and 7 [11]. A group of untreated rats was included to compare. All litters included MSG-treated and intact rats, which and stayed with the females until PD21. After that, animals were separated into subgroups of 4 animals per cage and kept under the bioterium conditions described above until PD57-60. Experiments were designed to minimize the suffering in the animals and the number of used animals. Animal care and experimental procedures were in accordance with the Mexican Official Norms NOM-062-ZOO-1999 and NOM-033-ZOO-1995, and also with the Directive 2010/63/EU referenced rules. Full experimental protocol was approved by the local Committee of Bioethics.

### Western blotting

At PD60, animals were euthanized by decapitation, their brain was extracted, and whole hippocampi (Hp) and entorhinal cortices (EC) were dissected out. Samples were immediately weighed and frozen at −20 °C for 24–72 h. Total protein content was extracted from the samples through homogenizing the tissue samples by sonication in lysis buffer (10 mM Tris–HCl pH 7.5, 150 mM NaCl, 20 mM NaF, 0.5 mM Na_3_VO_4_, and 1% NP40) with a protease inhibitor cocktail (Cat. sc-29130, Santa Cruz Biotechnology, CA, USA) at 4 °C. For each 100 mg of tissue, 920 μL of lysis buffer and 80 μL of protease inhibitor cocktail were added. Homogenates were centrifuged at 16,060 × *g* at 4 °C for 30 min, and the supernatants were recovered and stored at −20 °C. The protein concentration in the supernatants was determined by the Lowry method [59] (Bio-Rad, DC Protein Assay kit, Cat. 5000116, Bio-Rad Laboratories, CA, USA) with bovine serum albumin (Cat. 500–0007, Bio-Rad Laboratories, CA, USA) as the external standard. Sixty μg of total protein were denatured in 5 μL of Laemmli buffer (500 mM Tris–HCl pH 6.8, 2% SDS, 10% glycerol, 10% beta-mercaptoethanol, 0.1% bromophenol blue) at 95 °C for 5 min. Denatured proteins were electrophoresed in 10% SDS-PAGE gels with Tris-Glycine as the running buffer (25 mM Tris–HCl, 192 mM glycine, 0.1% SDS, pH 8.3; Cat. 1610723, Bio-Rad Laboratories, CA, USA), applying 110 V for 2 h. Then, electrophoresed proteins were blotted onto nitrocellulose membranes (Cat. 1620115, Bio-Rad Laboratories, CA, USA) in a wet system at 110 V for 0.5 h using a transfer buffer containing methanol (25 mM Trizma Base, 250 mM Glycine, 20% methanol, pH 8.8). Both electrophoresis and blotting were performed in a Mini-Protean Tetra Cell (Cat. 1658005, Bio-Rad Laboratories, CA, USA) using a PowerPac HC (Cat. 1645052, Bio-Rad Laboratories, CA, USA) as a power supply. To confirm the blotting efficiency we stained acrylamide gels with Bio-Safe Coomassie G-250 Stain solution (Cat. 1610786, Bio-Rad Laboratories, CA, USA) and nitrocellulose membranes with Ponceau S solution (Cat. P7170-1 L, Sigma-Aldrich Co., WI, USA), only those membranes without bubbles or without problems in blotting were processed to immunolabeling.

Blotted proteins in nitrocellulose membranes (Cat. 10600003, Amersham Protran, GE Healthcare Life Science, WA, USA) were incubated in 5% BLOT-QuickBlocker Reagent (Cat. WB57, EMD Millipore, MA, USA) in PBS-0.1% Tween 20 (PBST) for 30 min. Then, the membranes were washed 5 times in PBST for 3 min each, followed by incubation in primary antibodies: anti-NCX1 (1:10, Cat. sc-30304-R, Lot. G2914, Santa Cruz Biotechnology, CA, USA), anti-NCX2 (1:100, Cat. sc-33528, Lot. C1914, Santa Cruz Biotechnology, CA, USA), anti-NCX3 (1:25, Cat. sc-48896, Lot. H2013, Santa Cruz Biotechnology, CA, USA) and anti-β-actin (1:6000, Cat. ab 8227, Lot. GR40411-1, Abcam, Cambridge, UK) used as a reference protein. All primary antibodies were diluted in PBST-0.05% sodium azide and incubated for 18 h. Moreover to avoid contamination of immunolabeling each membrane was incubated only one time with only one primary antibody (not stripping of antibodies was done). Then, membranes were washed as described above and incubated in the respective secondary anti-body: HRP-goat anti-rabbit IgG for anti-NCX1 and anti-β-actin (1:7500 and 1:10000, respectively, Cat. 92680011, Lot. C30-118.03, LI-COR Bioscience, NE, USA) and HRP-chicken anti-goat IgG for anti-NCX2 and anti-NCX3 (1:5000, Cat. sc-2953, Lot. B2414, Santa Cruz Biotechnology, CA, USA). Secondary antibodies were diluted in PBS and incubated for 2 h. After this incubation, membranes were washed in PBS 5 times for 3 min each. All incubations described above were performed at 4 °C with continuous shaking. Finally, membranes were incubated in Western Sure Premium Chemiluminescent Substrate (Cat. 92695000, LI-COR Bioscience, NE, USA) at room temperature with shaking for 5 min. The chemiluminescent signal was acquired through a C-DiGit Blot Scanner (Cat. 6536–030, LI-COR Bioscience, NE, USA) and analysed through free Image Studio Lite Software 3.1.4 (LI-COR Bioscience, NE, USA).

The relative expression levels of the NCXs were calculated considering the ratio of the signal of each NCX band to the signal of corresponding β-actin. Data represent the mean ± SEM of five samples by group and cerebral region, which were analysed in triplicate.

### Animal stereotaxic surgery

Young adult rats (PD57-59) were anesthetized with an intraperitoneal mix of ketamine (90 mg/kg; Lot. C15J044, PiSA Pharmaceutical, Mexico) and xylazine (Lot. A084121, 15 mg/kg, PiSA PiSA Pharmaceutical, Mexico) and placed in a stereotaxic frame (Cat. MD3000, Basi Analytical Instruments, IN, USA) over a heating pad to maintain body temperature at 37 ± 2 °C during surgery with the incisor bar positioned 5 mm dorsal to the interaural line. According to Paxinos and Watson [60] and considering Bregma point as a reference, one stainless steel guide cannula (22 Gauge and 13 mm of length) was settled in the right lateral ventricle (RLV; Coordinates in mm: Anteroposterior (AP): −0.8, Lateral (L): −1.4, and Ventral (V): −0.5), and four copper electrodes (250 μm of diameter) were positioned in the right and left dorsal hippocampal CA1 region (Coordinates in mm: AP: −3.3, L: ±2, and V: −2.6) and in the right and left entorhinal cortex (Coordinates in mm: AP: −6.3, L: ±4.6, V: −8). As reference electrodes, two stainless steel screws with a copper wire welded, were placed on the left and right frontal sinus (Coordinates in mm: AP: 4, L: ±2, V: −0.6). Finally two screws were placed as support for the electrodes (Coordinates in mm: AP: −8, L: ±3, V: −0.6). All electrodes were soldered to a pluggable multipin connector. Dental acrylic cement was used to fix all elements to the rat skull.

### EEG recording

Three days after surgery, awake animals were connected to a Grass Polygraph (AC amplifiers Model 7P511J, Grass Medical Instrumentations, MA, USA), and EEG activity was digitalized and acquired through a USB1208FS device (MC Measurement Computing, MA, USA) controlled by a software designed in LabVIEW2014 (Vi platform, 32 bits; National Instruments, TX, USA) with similar characteristics to PolyVIEW software purchased by Grass-Telefactor. This acquisition system was calibrated with an external generator and programed to filter unspecific signals in 60–60.5 Hz (corresponding to electrical installation signal) and in 0.5 Hz (related to movement artefacts). The signal acquired with the amplifiers off had a net amplitude of 0.015 ± 0.003 mV and a frequency of 52.86 ± 12.18 Hz in general for all channels recorded; with the amplifiers on and unconnected animal had a net amplitude of 0.163 ± 0.017 mV and a frequency of 88.35 ± 0.00004 Hz; while with the amplifier on and control animals connected had a net amplitude of 0.899 ± 0.172 mV and a frequency of 1.831 ± 0.272 Hz in channels where Hp activity was recorded; whereas in channels where CE activity was recorded the net amplitude was 0.615 ± 0.090 mV and the frequency was 1.977 ± 0.256 Hz. Data of basal EEG activity represent mean ± SDM of twelve experiments for each experimental group and demonstrate that signal to noise ratio of our system in amplitude is more than the minimal ratio required (3:1).

During each experiment, 10 min of basal activity, followed by 5 min during i.c.v. infusion of 4-AP or KB-R7943 (alone or in combination), and 60 min after drug administration were acquired through EEG.

Because a very few studies have analysed the *in vivo* effects of KB-R7943, several doses of the NCXs blocker (500, 250, 125 and 62.5 pmol) were characterized at the beginning of this study. We selected 62.5 pmol as the dose of KB-R7943 to be used for more broad characterization. Then, control and MSG-treated rats received one of the following treatments: 1) 4-AP (3 nmol), 2) KB-R7943 (62.5 pmol) and 3) 4-AP (3 nmol) plus KB-R7943 (62.5 pmol). The 4-AP was purchased from Sigma-Aldrich, MO, USA (Cat. 504-24-5), and both stock (5 mg/mL) and infusion solutions (3 nmol/5 μL) were dissolved in 0.9% NaCl solution. KB-R7943 was purchased from Tocris Bioscience, Bristol, UK (Cat. 1244), stock solution (0.5 mg/mL) was dissolved in DMSO at 100 mM and infusion solution (62.5 pmol/5 μL) was dissolved in 0.9% NaCl solution. Infusion solutions were administered through the cannula (a dental injection needle 27 Gauge with 16 mm of length), which penetrated into infusion guide to reach the RVL (Coordinates in mm relative to Bregma: AP 0.8, L: −1.4, and V: −3.6). The cannula was connected to a microsyringe (Cat. ILS500TLL, WPI, FL, USA) installed in a microinjection pump (Model SP101L, WPI, FL, USA) to establish the infusion rate in 1 μL/min for 5 min. Direct blue 15 (Cat. D2535, Sigma-Aldrich, MO, USA) was added to the infusion solutions (5 mg/mL) to visual check the penetration of the drugs. At the end of the EEG recordings, animals were euthanized by decapitation, the brain was dissected, and implantation and infusion locations were verified; only animals with implants and infusions in the correct locations were included in our analysis.

The latency, duration and net amplitude of the first burst triggered by infusion solutions were analysed, and ictal activity duration and the elapsed time to EEG activity normalization were estimated. We considered as burst to electroencephalographic pattern characterized by continuous discharges with a predominant delta rhythm whose net amplitude was three-fold higher than the average basal activity and whose long was 30 s or more. To estimate ictal activity duration, in addition to burst activity, we considered continuous discharges patterns of short duration, peak-wave complexes, or isolated peaks with net amplitude at least two-fold higher than the average of basal activity (Table 1). The results are expressed as the mean ± SEM of 4 animals for each i.c.v. treatment in each experimental group.

**Table 1:** Basal EEG activity in studied cerebral region and groups

Parameter	Region	Control Group	MSG Group	*p* value
Net Amplitude (mV)	Hp	0.899 ± 0.172	0.664 ± 0.099	<0.001
	EC	0.615 ± 0.090	0.517 ± 0.104	0.023
Avarage Frequency (Hz)	Hp	1.831 ± 0.272	2.351 ± 0.806	0.046
	EC	1.977 ± 0.256	2.558 ± 0.854	0.034

*Hp* hippocampus, *EC* entorhinal cortex

Values represent mean ± SDM of twelve animals per group. *p* values were obtained through the Student’s *t*-test with 95% confidence interval

### Statistical analysis

The statistical analysis was performed using Student’s *t*-test for the western blot analysis, basal EEG activity and body weight comparisons, Mann–Whitney U test for bursts number comparison, and a two-way ANOVA with Tukey’s *post hoc* test for the comparison of the other variables measured through EEG recordings. Statistical significance was set at *p* < 0.05 using the SPSS version 20.0 (SPSS, IBM Analytics, NY, USA).

## Results

### Expression of NCX1-3 proteins in the EC and Hp

All three types of NCX were detected in our samples with only one specific band for NCX2 (102 kDa) and more than one for NCX1 and 3, but only the bands equivalent to 67 and 66 kDa for NCX1 and NCX3, respectively, displayed a specific relationship between the signal and protein concentration and a higher signal than the other bands. No consistent bands over 100 kDa were detected with the NCX1 and NCX3 antibodies used here (Additional file 1). The other bands observed for NCX1 and NCX3 may correspond to some isoforms reported previously for these exchangers [28, 61, 62].

In the control group, at PD60, the NCX2 expression levels were higher than that of NCX1, followed by the expression levels of NCX3, in both the EC and Hp, with higher values observed in the EC. MSG treatment did not modify the relationship described above.

**Fig. 1:**
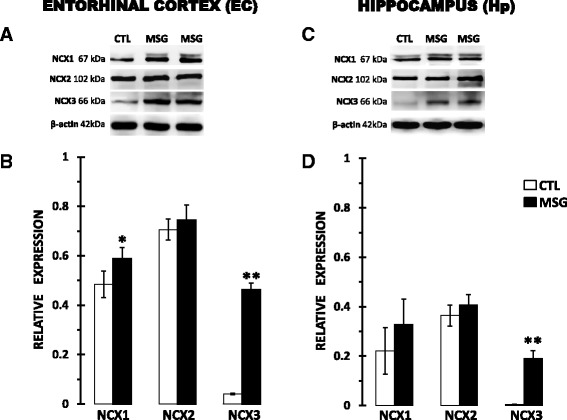
Neonatal MSG treatment effects on NCX1-3 protein expression in the entorhinal cortex (*left*) and hippocampus (*right*) at PD60. Upper panels **a** and **c** correspond to representative images of western blots for each analysed protein in control (CTL) and MSG-treated rats (MSG). Lower panels **b** and **d** correspond to graphic representations of the relative expression of each NCX protein with respect to β-actin expression. The colourless bars correspond to the CTL group, and the dark bar to the MSG group. Values represent the mean ± SDM from five animal samples for each cerebral region and group analysed in triplicate. Statistically different at *p < 0.05 and **p < 0.01 for the MSG compared to the CTL group using a Student’s *t*-test

Neonatal MSG treatment significantly increased the expression levels of NCX1 (*p* < 0.05) in the EC and NCX3 in both the EC and Hp (*p* < 0.01) measured at PD60. The expression level of the NCX2 protein was also slightly augmented in both cerebral regions studied at PD60 after treatment, but the change was not significant (Fig. 1).

### KB-R7943 effects on the electrographic and seizure activity in the EC and Hp

Since neonatal MSG treatment modified the expression level of NCX1 and NCX3, we decided to evaluate the effects of i.c.v. infusion of KB-R7943. However to identify clearly KB-R7943 or 4-AP effects on EEG activity, in each experiment we first analysed the effect of MSG treatment exerted on basal activity, we noted that MSG treatment reduced net amplitude and increased average frequency in both the EC and Hp (Table 1).

**Fig. 2:**
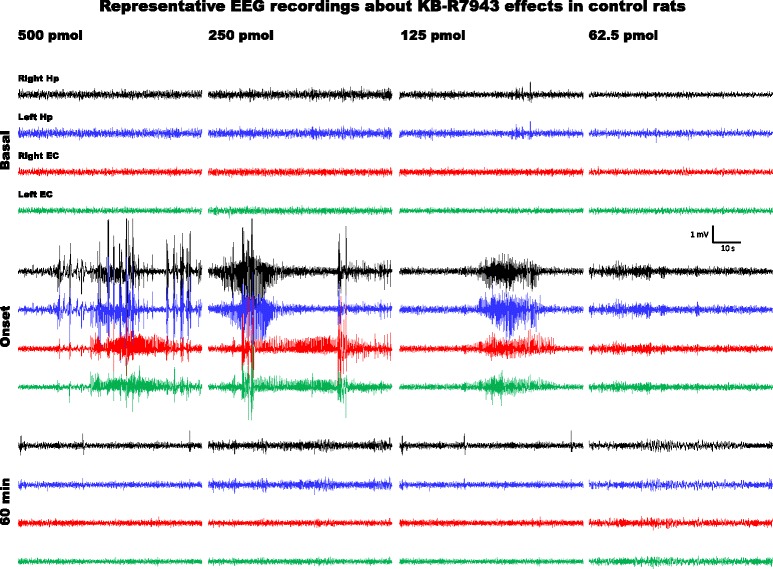
EEG activity generated in the entorhinal cortex and hippocampus of control rats (CTL group) at PD60 by i.c.v. infusion of KB-R7943 at different doses. Representative one-minute fragments from the recordings taken during basal activity (5 min before the infusion), at the onset of altered activity, and 1 h after the infusion was completed are presented for 500, 250, 125 and 62.5 pmol of KB-R7943

Because there are no previous studies that have administered this isothiourea into the brain; therefore, we evaluated several doses between 500 to 62.5 pmol. The highest dose of KB-R7943 (500 pmol) induced bursts and like-epileptiform activity in the EEG recordings of the cerebral regions studied (Fig. 2) as well as convulsive behaviours, such as facial clonus, chewing, salivation, head clonus and wild running. The lowest KB-R7943 dose (62.5 pmol) only induced slight changes in EEG recordings, but no bursts (Fig. 2) or major behavioural changes were observed (Table 2). Therefore, we decided to use 62.5 pmol of KB-R7943 to evaluate the effects of blocking NCXs on seizures induced by 4-AP.

**Table 2:** Features and epileptiform behaviours observed in the studied groups

Group	Weight (g)	Subgroups	Bursts	Behaviours	Death
CTL	254.99 ± 20.03	4-AP	1–3	facial, head and forelimb clonus, wild running and rearing	Not observed
		KB-R7943	0-2^b,c^	clonic convulsions of head	Not observed
		4-AP + KB-R	0-4^b,c^	clonic convulsions of head	Not observed
GMS	164.53 ± 10.70^a^	4-AP	6-11^b^	facial, head and forelimb clonus and GTCS	Observed in 1 animal
		KB-R7943	0-1^b,c^	facial clonus	Not observed
		4-AP + KB-R	0-1^b,c^	facial clonus	Not observed

^a^ *p* < 0.01 CTL vs. MSG animals, using Student’s *t*-test; ^b^ *p* < 0.05 all subgroups compared to CTL/4-AP, and ^c^ *p* < 0.05 all subgroups compared to MSG/4-AP, both using Mann–Whitney U test. Doses: 3 nmol of 4-AP and 62.5 nmol of KB-R7943 (also named KB-R)

**Fig. 3:**
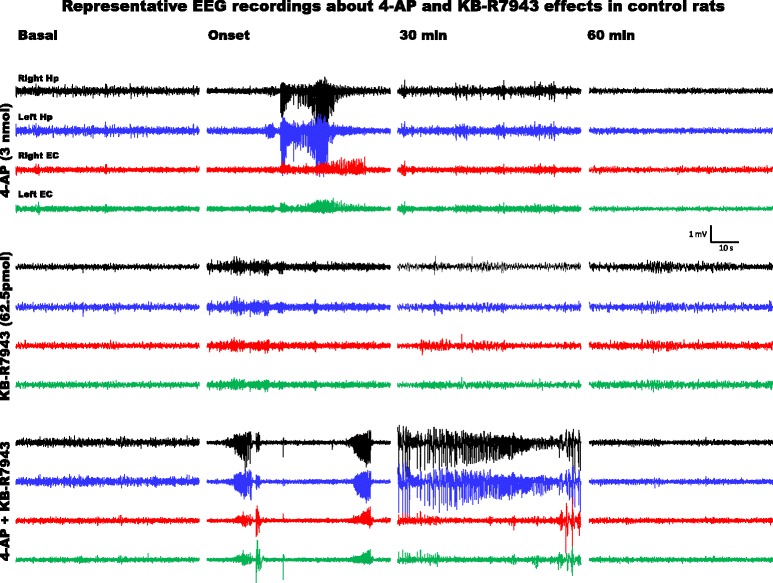
EEG activity generated in the entorhinal cortex and hippocampus of control rats (CTL group) at PD60 with i.c.v. infusion of 4-AP (3 nmol), KB-R7943 (62.5 pmol) or the combination of 4-AP (3 nmol) and KB-R7943 (62.5 pmol). Representative one-minute fragments from the recordings taken during basal activity (5 min before the infusion), at the onset of altered activity, and 30 and 60 min after completion of the infusion are presented

**Fig. 4:**
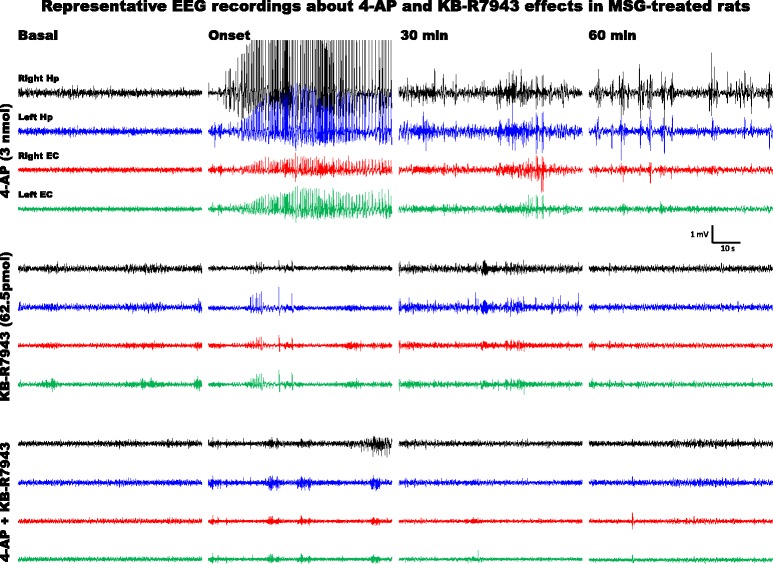
EEG activity generated in the entorhinal cortex and hippocampus of MSG-treated rats at PD60 with i.c.v. infusion of 4-AP (3 nmol), KB-R7943 (62.5 pmol) or the combination of 4-AP (3 nmol) and KB-R7943 (62.5 pmol). Representative one-minute fragments from the recordings taken during basal activity (5 min before the infusion), at the onset of altered activity, and 30 and 60 min after completion of the infusion are presented. KB-R7943 significantly reduced epileptiform activity induced by 4-AP in MSG-treated rats

According to previous studies from our group, i.c.v. infusion of 3 nmol of 4-AP was applied to induce epileptiform activity. In control rats, this 4-AP dose generated 1–3 EEG bursts (Fig. 3) and facial, head and forelimb clonus followed by more severe convulsive behaviour such as wild running and rearing (Table 2). In contrast, in MSG-treated rats, 3 nmol of 4-AP induced 6–11 EEG bursts (Fig. 4) and generalized tonic-clonic seizures (GTCS) along with more severe convulsive behaviour (Table 2). KB-R7943 alone or mixed with 4-AP increased the number of EEG bursts (Fig. 4), and convulsive behaviour was confined to minimal clonic convulsions of head (Table 2), whereas in MSG-treated rats, KB-R7943 alone or mixed with 4-AP reduced the number of EEG bursts (Fig. 4) and the convulsive behaviour to facial clonus (Table 2).

**Fig. 5:**
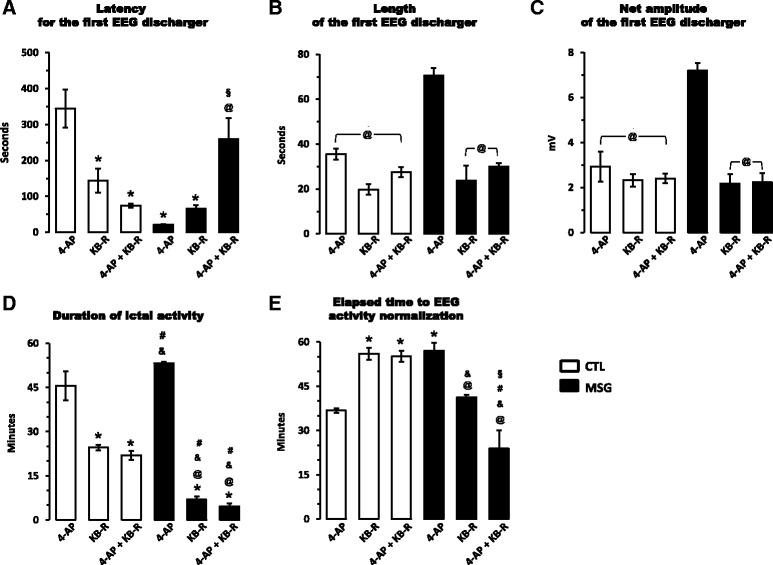
KB-R7943 (62.5 pmol) effects on epileptiform EEG activity induced by 4-AP (3 nmol) in control (*colourless bars*) and MSG-treated (*black bars*) rats. The latency, duration and net amplitude of the first burst recorded in the right hippocampus after i.c.v. infusion of the drugs alone or in combination are presented in panel **a**, **b** and **c**, respectively. Furthermore, the duration of ictal activity **d** and elapsed time before EEG activity normalization **e** were also measured in the same conditions described before. Activity recorded in the right hippocampus was considered for these measurements because it was the region closest to the infusion location. Data represent the mean ± SEM from four animals for each infusion scheme (subgroups: 4-AP, KB-R7943, and 4-AP + KB-R7943) in each group (CTL and MSG). Statistically different at *p < 0.05 for all subgroups compared to that in control rats with 4-AP; @*p* < 0.05 for all subgroups compared to that in MSG-treated rats with 4-AP; &*p* < 0.05 for all subgroups compared to that in control rats with KB-R7943; #*p* < 0.05 for all subgroups compared to that in control rats with 4-AP + KB-R7943; and §*p* < 0.05 between KB-R7943 alone and 4-AP + KB-R7943 in MSG-treated animals; tested using two-way ANOVA with *post hoc* Tukey’s test. Furthermore, *p* and *F* values, including degree of freedom, for each variable compared by two-way ANOVA are summarized in Additional File 2

To clarify the effects of KB-R7943 on seizure susceptibility, we decided to analyse the electrographic parameters of the first EEG burst recorded in the right Hp at the level of the CA1 (the recorded area most proximal to the infusion location). In control rats, the latency of the first burst was close to 6 min after 4-AP infusion, and it was shorter after KB-R7943 or KB-R7943 + 4-AP infusion. In MSG-treated rats, the latency of the first burst was close to 20 s after 4-AP infusion and it was longer after infusion of KB-R7943 or KB-R7943 + 4-AP (Fig. 5a). Furthermore, the first burst induced by 4-AP was longer (Fig. 5b) and higher (Fig. 5c) in MSG-treated rats than in controls, and KB-R7943 significantly reduced both parameters when it was applied with 4-AP (Fig. 5b, c).

On the other hand, the duration of ictal activity induced by 4-AP was slightly but not significantly longer in MSG-treated rats than in the controls, and KB-R7943 induced a significant reduction in this parameter, in both control and MSG-treated rats, where the reduction was close to 90% (Fig. 5d). The time elapsed until the normalization of EEG activity (return to basal parameters) was longer in control rats after KB-R7943 and KB-R7943 + 4-AP infusion and also in the MSG-treated rats after 4-AP infusion than in the controls infused with only 4-AP. Moreover this parameter was significantly reduced by KB-R7943 infused in combination with 4-AP (Fig. 5e).

Statistical analysis of electrographic parameters mentioned above applying two-way ANOVA test to evaluate the interactions between the treatments (Main treatment: Groups: Control and MSG; and Conditional treatment: Subgroups: 4-AP, KB-R7943 and 4-AP + KB-R7943) showed that they (Treatments: Groups/Subgroups) have a significant interaction on all evaluated parameters (see Additional file 2). Furthermore, to identify clearly the effect of each combination between treatments (Control/4-AP; Control/KB-R7943; Control/4-AP + KB-R7943; MSG/4-AP; MSG/KB-R7943; and MSG/4-AP + KB-R7943) a *post hoc* Tukey’s test were applied and founded significant differences are showed in the Fig. 5.

## Discussion

Neonatal MSG treatment induced long-term upregulation of NCX1 and NCX3 protein expression and modified the functional implication of these exchangers in the control of seizure susceptibility. In a previous work, increases in the expression level of mRNA and protein of NCX3 were already observed at PD8 in the Hp after MSG treatment [19]. Here, NCX3 protein expression was augmented by MSG treatment in both the Hp and EC at PD60, which suggests that the upregulation of NCX3 expression could be a long-term effect of the treatment and also that this outcome may be present in other cerebral regions; however, these hypotheses should be tested yet. In this sense, a developmental profile of the NCXs expression should be established for both the EC and Hp in both controls and MSG-treated rats in future studies.

Because NCX3 expression has been previously reported to be lower than that of NCX1 and NCX2 [30, 31], the increase induced by MSG treatment may be even more relevant for the physiology of the studied regions, where NCX3 is classically confined to the neurons at the soma level and dendrites and is found to be strongly expressed in mossy fibres projecting to the CA3 in the Hp and in layers V and VI of the EC [30, 33, 63]. In contrast, high expression levels of NCX1 in the Hp are confined to the distal dendrites [33] and to layer II in the EC [63]. MSG treatment significantly increased only NCX1 expression in the EC at PD60; no similar change has been reported previously in other excitotoxicity models. Furthermore, although neonatal MSG treatment induces neuronal loss [9–11], it also increases glial cell density [12], where NCX1 and NCX3 may be expressed [29, 33, 64]; however, analysis of the immunochemical location of these proteins must be performed to confirm this possibility.

Due to the crucial role of NCX exchangers in Ca^2+^ homeostasis, several studies have tried to clarify their involvement in the excitotoxicity process. *In vitro* studies have demonstrated that activation of the NMDA receptor under glucose deprivation increases [Ca^2+^]_i_ and promotes NCX reversal activity, prolonging the Ca^2+^ overload and enhancing neuronal death [65]. Moreover, the increase in [Ca^2+^]_i_ and reversal of NCX activity stimulates calpains, which act on NCX3 and lead to loss of its activity, followed by a permanent high [Ca^2+^]_i_ [2, 6, 65]. Proteolysis of NCX3 by calpains specifically produces fragments of low molecular weight proximate to 60 kDa [2, 6, 66], possibly corresponding to the bands observed in our western blot analysis for this protein. NCX1 and NCX2 have been reported to be resistant to proteolysis induced by calpains [2, 6, 66]. Proteolysis of NCX3 has also been observed *in vivo* after ischaemia secondary to middle cerebral artery occlusion (MCAO) [2, 67]. Thus, most of the *in vivo* studies have reported reductions in the expression level of NCXs as a consequence of an excitotoxic process, which differs of our results. In chronic epileptic adult rats, the expression of the NCX1 protein was decreased in the dentate gyrus and layer III of the EC; similarly, NCX3 protein expression was diminished in the *stratum lucidum* and hilar region of the dentate gyrus, and both reductions were permanent from 3 weeks until 2 months after the induction of *status epilepticus* by kainic acid and were closely related to neuronal death in the same regions [63]. Seizures induced by hyperthermia in young rats (PD20) have been related to downregulation of NCX3 expression in the Hp and cerebral cortex [68], while seizures induced by pentylenetetrazol in adult mice decrease NCX1 and NCX2 expression in the Hp without modifications in NCX3 expression [69]. In adult rats, ischaemia reduces the expression of NCX1 and NCX3 without affecting NCX2 in the temporoparietal cortex, but when ischaemic preconditioning or postconditioning are applied, the expression levels of NCX1 and NCX3 are augmented, with more significant effects at 24 h of reperfusion [70, 71]. Although it is unknown if the increases produced by ischaemic preconditioning or postconditioning remain, it is possible that repeated administration of MSG in the treatment scheme applied here acts as postconditioning and upregulates the expression of NCX1 and NCX3 in the long term; however, this statement should be analysed.

Several intracellular signalling pathways have been associated with the regulation of NCX expression. Overexpression of NCX1 and NCX3 induced by a short exposure to NGF is dependent on activation of the p38 and ERK1/2 signalling pathways [27]. Similarly, neuronal death induced by neonatal MSG treatment in the cerebral cortex [9] and Hp [10] is dependent on activation of the p38 signalling pathway. Moreover, neonatal MSG treatment produces long-term positive effects through activation of the ERK1/2 signalling pathway measured in the hypothalamus [72]. Then, both the p38 and ERK1/2 signalling pathways could be related to the long-term increase in NCX1 and NCX3 expression observed here after MSG treatment, but it remains to be demonstrated.

Functionally, expression of NCX1 and NCX3 plays an important role in cell survival and is positively regulated by growth factors such as NGF [27] and BDNF [25] in a way that increases neural differentiation and reduces neuronal death by excitotoxicity, respectively. NCX3 overexpression has been related to seizure resistance [73] and downregulation of seizure susceptibility [68, 73]. Evidence on the roles of NCX1 is contradictory, as NCX1 knock-out mice are resistant to seizures induced by pentylenetetrazole [74], but NCX1 downregulation has been related to epileptogenesis in a kindling model [69]. Here, even though MSG-treated rats show an augmented expression of NCX3 and NCX1, they are more susceptible to seizures induced by 4-AP [44], probably because the changes in the expression of NCXs are accompanied by a very broad and complex set of modifications [18, 25], including reductions in GABAergic inhibitory neurotransmission [14, 15]. Then changes in NCXs described above are only a part of the mechanisms implicated in the process by which MSG-treated rats are more susceptible to seizures induced by 4-AP [44]. Furthermore, because neural depolarization may induce inversion of NCXs and the inversion may increase firing probability [75, 76], changes induced by MSG treatment as increased average frequency of basal EEG activity in both studied cerebral regions (reported here for first time; Table 1), suggest that the probability of NCXs inversion could be potentiated after the treatment; however this suggestion should be proved.

In our study, KB-R7943 reduced the duration of ictal activity induced by 4-AP in both control and MSG-treated rats (Fig. 5d), suggesting a general effect of NCXs blocking on control of seizure propagation and generalization. However, only in MSG-treated rats the KB-R7943 increased the latency, and reduced the length and net amplitude of the first burst induced by 4-AP (Fig. 5a-c), suggesting that after neonatal MSG treatment the blockage of NCXs in adult rats delay the onset of seizures. Furthermore, convulsive behaviours induced by 4-AP were reduced by KB-R7943 in both experimental groups (Table 2). Then, although action mechanisms of KB-R7943 are still controversial, and it seems to block not only NCXs but also other transporters and receptors [77–80], we proposed that the effects exerted by KB-R7943 on seizure control in MSG-treated rats are related at least in part, to the increases in expression level of NCX1 and NCX3 described above.

On the other wise, *in vitro* approaches have demonstrated that low concentrations (<1 μM) of KB-R7943 predominantly block the reverse mode of NCX3 [81], avoiding the rise in [Ca^2+^]_i_ and reducing the frequency of excitatory postsynaptic currents, but high concentrations (≥10 μM) can block both the reverse and forward modes of the NCXs and promote increases in both [Ca^2+^]_i_ and the frequency of excitatory postsynaptic currents [76]. Because very few studies have applied KB-R7943 intracerebrally, we are not able to specifically propose how KB-R7943 is exerting its effects on the seizures. However, since all KB-R7943 doses proved here (500–62.5 pmol) in control rats induced alterations on basal EEG activity, and 62.5 pmol of KB-R7943 in both experimental groups induced similar EEG alterations; we propose that when this isothiourea is applied alone, then predominantly blocks the forward mode of NCXs. In contrast, when KB-R7943 was applied in combination with 4-AP, it is possible that reverse mode blocking happen, since prolonged neural depolarization induces inversion of NCXs [65, 67, 75]. In addition, if we take in count that in MSG-treated rats: 1) neural inhibition mediated by GABA is reduced [14, 15, 44] and 2) NCX1 and NCX3 are overexpressed; then it is possible that the NCXs inversion may be reached sooner than in control rats; however more studies are necessary to clarify these statements.

Since a previous work demonstrated that SEA0400, a specific blocker of NCX1, did not have a significant effect on 4-AP induced seizures [74], it is possible that exerted effects of KB-R7943 on control seizures induced by 4-AP mainly depends on the blockage of NCX3, which should be analysed in future studies.

Finally, because the beneficial effects of KB-R7943 on seizure control after a degenerative process triggered by neonatal MSG treatment are clear, we suggest that the blockage of NCXs as possible therapeutic target for epilepsies developed after an excitotoxic process, which must be analysed more broadly.

## Conclusions

This is the first study reporting long-term effects of an excitotoxic process on functional expression of NCXs in the EC and Hp. In particular, neonatal MSG treatment increased the expression of NCX1 only in the EC and NCX3 in both of the studied regions at PD60. Furthermore, the changes induced by the intracerebral administration of KB-R7943 on EEG activity and behaviour of adult rat also were described here for first time. Specifically, KB-R7943 generated EEG discharges and behaviours similar to epileptiform activity in more significant way in control rats; but when it is administered in combination with 4-AP, KB-R7943 control the seizures, in more significant way in MSG treated rats. Thus, according to KB-R7943 selectivity, our results suggest that the role of NCXs, and particularly of NCX3, should be better characterized in the changes induced in seizure susceptibility produced by an excitotoxic process.

## Additional files

Additional file 1: Representative images with the complete banding pattern are showed in each panel for samples of total protein extract obtained of the hippocampus of adult rats. (PPTX 315 kb)

Additional file 2: Results of two-way ANOVA test (in *p* values) applied to determinate the interactions between treatments on the electrographic parameters evaluated in this work. (DOCX 16 kb)

## Abbreviations

Hp: Hippocampus
EC: Entorhinal cortex
MSG: Monosodium glutamate
EEG: Electroencephalogram
4-AP: 4-aminopyridine
KB-R7943: (2-[2-[4-(4-nitrobenzyloxy)phenyl]ethyl] isothiourea methanesulfonate)
i.c.v.: Intracerebroventricular
s.c.: Subcutaneous
i.p.: Intraperitoneal
