# Supplementary material for: KB-R7943 reduces 4-aminopyridine-induced epileptiform activity in adult rats after neuronal damage induced by neonatal monosodium glutamate treatment
Source: J Biomed Sci. 2017 May 9;24:27. doi: 10.1186/s12929-017-0335-y (PMC5423021; doi:10.1186/s12929-017-0335-y)
Supplement: Supplementary file 1 — Representative images with the complete banding pattern are showed in each panel for samples of total protein extract obtained of the hippocampus of adult rats. (PPTX 315 kb) [file 12929_2017_335_MOESM1_ESM.pptx]

## Slide 1
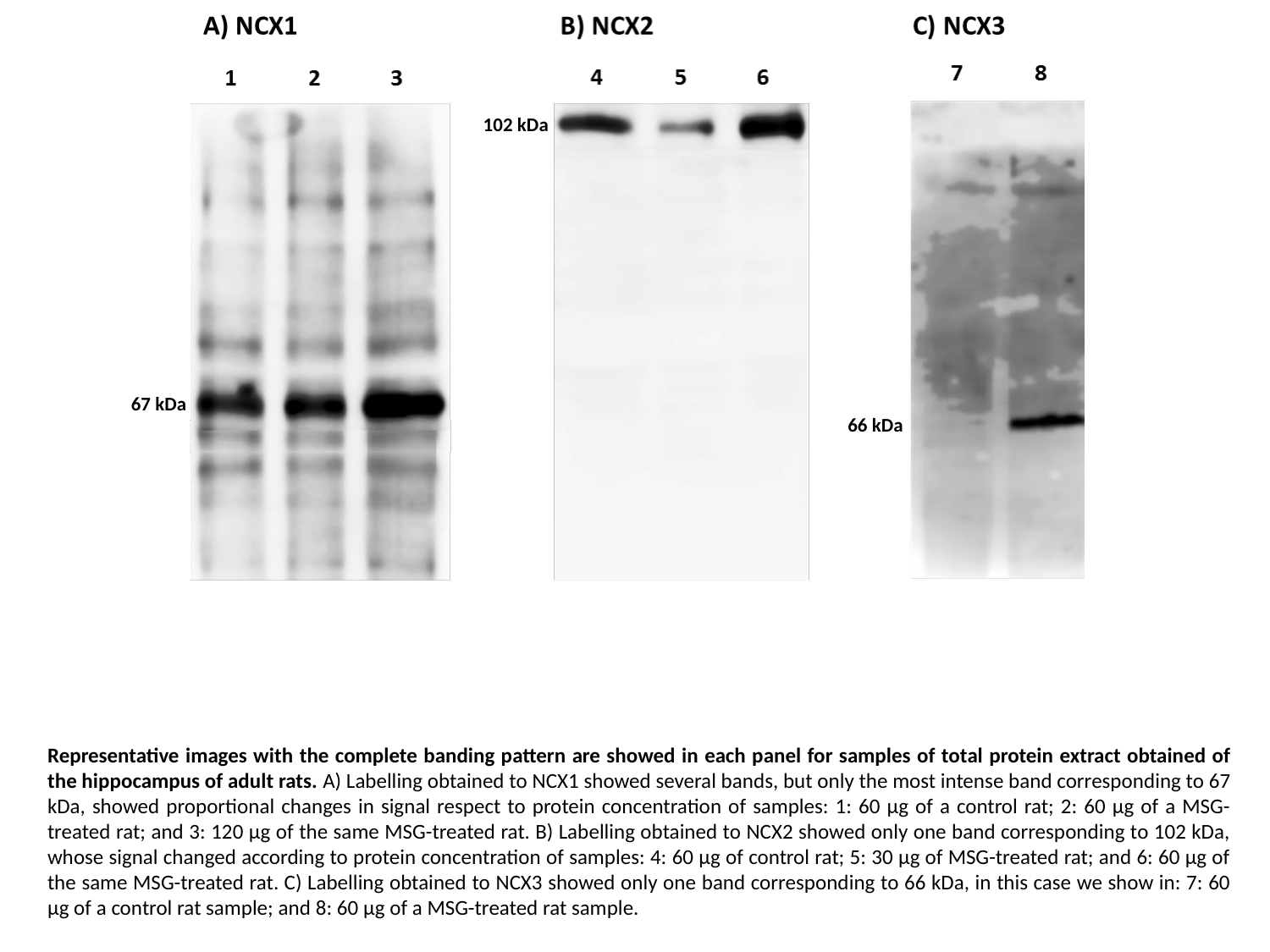

102 kDa
67 kDa
66 kDa
Representative images with the complete banding pattern are showed in each panel for samples of total protein extract obtained of the hippocampus of adult rats. A) Labelling obtained to NCX1 showed several bands, but only the most intense band corresponding to 67 kDa, showed proportional changes in signal respect to protein concentration of samples: 1: 60 µg of a control rat; 2: 60 µg of a MSG-treated rat; and 3: 120 µg of the same MSG-treated rat. B) Labelling obtained to NCX2 showed only one band corresponding to 102 kDa, whose signal changed according to protein concentration of samples: 4: 60 µg of control rat; 5: 30 µg of MSG-treated rat; and 6: 60 µg of the same MSG-treated rat. C) Labelling obtained to NCX3 showed only one band corresponding to 66 kDa, in this case we show in: 7: 60 µg of a control rat sample; and 8: 60 µg of a MSG-treated rat sample.
