# Supplementary material for: KB-R7943 reduces 4-aminopyridine-induced epileptiform activity in adult rats after neuronal damage induced by neonatal monosodium glutamate treatment
Source: J Biomed Sci. 2017 May 9;24:27. doi: 10.1186/s12929-017-0335-y (PMC5423021; doi:10.1186/s12929-017-0335-y)
Supplement: Supplementary file 2 — Results of two-way ANOVA test (in p values) applied to determinate the interactions between treatments on the electrographic parameters evaluated in this work. (DOCX 16 kb) [file 12929_2017_335_MOESM2_ESM.docx]

**Results of two-way ANOVA test (in *p* values) applied to determinate the interactions between treatments on the electrographic parameters evaluated in this work.**

| PARAMETER IN COMPARISON | COMPARISON LEVEL | | |
| --- | --- | --- | --- |
|  | **Groups** | **Subgroups** | **Groups/Subgroups** |
| Latency | *p* = 0.060404 | *p* = 0.201650 | *p* = 0.000077, *F* (2,18) = 16.780 |
| Length of discharger | *p* = 0.000385 | *p* < 0.000000 | *p* = 0.00358, *F* (2,18) = 12.734 |
| Net amplitude | *p* = 0.003434 | *p* = 0.000012 | *p* = 0.00192, *F* (2,18) = 14.289 |
| Duration of ictal activity | *p* = 0.000345 | *p* < 0.000000 | *p* = 0.00090, *F* (2,18) = 16.328 |
| Elapsed time to EEG activity normalization | *p* = 0.006514 | *p* = 0.019462 | *p* = 0.000001, *F* (2,18) = 32.671 |

NOTES: 1. The treatments were: Groups: Control and MSG; and Subgroups: 4-AP, KB-R7943 and 4-AP+KB-R7943.

2. In comparison Groups/Subgroups: *F* values and freedom degrees (in parentheses) were also included.
